# Transcriptional profiling of two muscadine grape cultivars “Carlos” and “Noble” to reveal new genes, gene regulatory networks, and pathways that involved in grape berry ripening

**DOI:** 10.3389/fpls.2022.949383

**Published:** 2022-08-11

**Authors:** Yuru Chang, Yogesh Kumar Ahlawat, Tongjun Gu, Ali Sarkhosh, Tie Liu

**Affiliations:** ^1^Department of Horticultural Science, University of Florida, Gainesville, FL, United States; ^2^Bioinformatics, Interdisciplinary Center for Biotechnology Research, University of Florida, Gainesville, FL, United States; ^3^Department of Biostatistics, University of Florida, Gainesville, FL, United States

**Keywords:** postharvest, stress tolerance, switch gene, non-climacteric fruit, ripeness

## Abstract

In commercial fruit production, synchronized ripening and stable shelf life are important properties. The loosely clustered or non-bunching muscadine grape has unrealized potential as a disease-resistant cash crop, but requires repeated hand harvesting due to its unsynchronized or long or heterogeneous maturation period. Genomic research can be used to identify the developmental and environmental factors that control fruit ripening and postharvest quality. This study coupled the morphological, biochemical, and genetic variations between “Carlos” and “Noble” muscadine grape cultivars with RNA-sequencing analysis during berry maturation. The levels of antioxidants, anthocyanins, and titratable acids varied between the two cultivars during the ripening process. We also identified new genes, pathways, and regulatory networks that modulated berry ripening in muscadine grape. These findings may help develop a large-scale database of the genetic factors of muscadine grape ripening and postharvest profiles and allow the discovery of the factors underlying the ripeness heterogeneity at harvest. These genetic resources may allow us to combine applied and basic research methods in breeding to improve table and wine grape ripening uniformity, quality, stress tolerance, and postharvest handling and storage.

## Introduction

Muscadine grapes [*Muscadinia rotundifolia* (Michx.) Small] are native to the southeast United States and are the first grape species to be cultivated in North America (Andersen et al., 2010; Hickey et al., 2019). Their natural habitat extends from central Florida to Delaware, along the Gulf of Mexico to Texas, and through the Mississippi River to Mississippi (Olien, 1990; Andersen et al., 2010). Muscadine grape taxonomy has been a controversial issue (Hickey et al., 2019). Once, the genus *Vitis* was characterized into two subgenera: *Euvitis* (bunch grapes) and *Muscadinia* (muscadine grapes; Lu et al., 1998). Recent molecular phylogenetic studies have revealed that *Vitis* and *Muscadinia* are two separated genera in the *Vitaceae* family (Cochetel et al., 2021). The most important genetic difference between *Vitis* and *Muscadinia* is the number of somatic chromosomes: *Muscadinia* has 2*n* = 40 chromosomes, while *Vitis* has 2*n* = 38 chromosomes (Campbell et al., 2021).

Muscadine grape shows some distinctive botanical features compared to bunching *Vitis* spp. Muscadine grapes grow in small and loose clusters instead of bunches, have a bronze- or purple-colored thick skin, and are adapted to warm and humid climates (Vasanthaiah et al., 2011). Muscadine grapes possess a large amount of phenolics, flavanols, anthocyanins, and display high antioxidant levels (Lee et al., 2005; Sandhu and Gu, 2010). Therefore, muscadine grapes show higher tolerance to many biotic and abiotic stresses (Louime et al., 2010). Muscadine grapes can be naturally resistant to a bacterium (e.g., Pierce’s disease caused by *Xylella fastidiosa*), viruses, phytoplasmas, nematodes, as well as some fungal diseases, such as downy mildew (*Plasmopara viticola*) and bunch rot (*Botrytis* spp.; Schoulties and Hopkins, 1978; Milholland, 1991; Hickey et al., 2019). The unique aroma and flavor as well as high sweetness levels make muscadine grape a favorite in local markets as a fresh fruit and are widely used for fermentation into wine and processing into juice, jam, jelly, or puree (Olien and Hegwood, 1990). Muscadine grapes are a good source of dietary fiber, polyphenols, and antioxidants, including anthocyanins, gallic acid, ellagic acid, catechin, epicatechin, quercetin, kaempferol, and resveratrol (Huang et al., 2009). These phytochemicals offer high antioxidant capacity and health benefits such as anti-inflammatory (Bralley et al., 2007), anti-oxidation, anti-microbial (Xu et al., 2014), anti-cancer (Balasubramani et al., 2019), and anti-cardiovascular disease (Pastrana-Bonilla et al., 2003) activities. Due to their health benefits, demand for muscadine wines has increased recently. There are over 100 improved muscadine grape cultivars. “Carlos” is a bronze- or light-skinned cultivar., while “Noble” is a purple- and dark-skinned cultivar (Xu et al., 2017). “Carlos” and “Noble” are the industry standards for juice and wine production because they possess the highest vigor, consistently produce the highest yield (roughly 45 kg/vine), and can produce a high-quality fruit (Carroll, 1986; Anderson, 2006). These cultivars are highly recommended because they are self-fertile, super vigorous, and productive. “Noble” tastes less musky and has better color retention, which are important characteristics for juice or wine industry (Anderson, 2006).

So far, limited research has been performed on the genes involved in formation of berry color (anthocyanin content) and the ripening process in *M. rotundifolia*. Nonetheless, the close link between *V. vinifera* and *M. rotundifolia* genomes has enabled the application of new genomic technologies and resources from *V. vinifera* to promote molecular genetic analysis in *M. rotundifolia*. A global grapevine gene expression atlas using microarray and RNA-sequencing (RNA-seq) analyses defines the sample transcriptomes into vegetative/green and mature/woody categories based on maturity and developmental stage rather than organ identity, revealing a fundamental transcriptomic reprogramming during the maturation process (Fasoli et al., 2012). Gene co-expression analysis further demonstrated the dynamic reprogramming of the transcriptome during maturation. The complete genome sequence combined with this comprehensive grape transcriptome map forms the foundation for global gene functional analysis of perennial fruit crops, elevating grape to the status of a model fruit species (Fasoli et al., 2012). As part of an integrated network analysis, the authors identified a category termed “fight-club hubs,” reflecting a negative correlation with the expression profiles of neighboring genes. A subset of these fight-club genes were named “switch genes,” which function as key regulators of the transcriptome reprogramming during maturation (Palumbo et al., 2014). The transcripts of all switch genes are at low levels in vegetative/green tissues, but increase significantly in mature/woody organs, indicating that they may play a regulatory role throughout the developmental transition (Palumbo et al., 2014).

The *V. vinifera* and *M. rotundifolia* genomes are substantially collinear, but do differ in chromosome number. Lewter et al. (2019) constructed the first genetic linkage maps using saturated genotyping-by-sequencing in two muscadine F1 populations segregating for flower sex and berry color. The muscadine linkage maps consisted of 20 linkage groups (LGs) for each F1 population. Except for linkage groups (LGs) 7 and 20, a high degree of collinearity was discovered between these genetic maps and the physical map of *V. vinifera*, implying a substantially conserved genome structure between the two species. The flower sex locus mapped on chromosome 2 in both *V. vinifera* and *M. rotundifolia.* Another noteworthy difference was detected in genes controlling fruit color. The MYB transcription factor genes that control fruit color were found on chromosome 2 in *V. vinifera*, but chromosome 4 in muscadine, implying that muscadine berry color is determined by a mutation in a different gene in the anthocyanin biosynthesis pathway and that the MYB transcription factor gene cluster on chromosome 2 is not a major predictor (Lewter et al., 2019).

Muscadine berry development is a complicated process involving a series of morphological, biochemical, physiological, and metabolic changes (Basiouny and Himelrick, 2002; Massonnet et al., 2017). During the bunch closure stage, the berries touch, and the cluster starts to close. At this stage, berries are green, highly acidic, and bitter due to the presence of chlorophyll, accumulation of organic acids, and high concentration of tannins in the skin (Conde et al., 2007). Veraison is defined as the transition to the ripening phase, while ripeness occurs when the seeds are mature. Berries soften and change color as they begin to ripen. During ripening, further metabolic changes make the berry edible, such as accumulation of sugar, loss of organic acids and tannins, and synthesis of volatile compounds (Conde et al., 2007). At the mature stage, black (deep purple) cultivars accumulate anthocyanins in the vacuoles of skin cells, but bronze (greenish yellow) or white cultivars do not because of mutations in *MYB* genes. The anthocyanin content in bronze muscadine skins is typically less than 100 μg g^−1^, while in dark, muscadine skins range roughly from 1,000 to 5,000 μg g^−1^ (Conner and MacLean, 2013). Purple muscadine grapes are more common in the wild, because bronze berry color is recessively inherited. The recessive allele is related to the insertion of Gret1, a single gypsy-type retrotransposon, into the promoter region of *VvMybA1*, three single-nucleotide polymorphisms, and one 2-bp insertion/deletion (Stuckey, 1919; Kobayashi et al., 2004; This et al., 2007; Fournier-Level et al., 2009). These polymorphisms result in structural changes in the MYB proteins and alternations in the *VvMybA*s promoters (Fournier-Level et al., 2009). *MYBA1* encodes a transcription factor required for anthocyanin synthesis and accumulation in red-skinned grape cultivars and is mutated and inactive in white-skinned cultivars (Massonnet et al., 2017). Many white grape cultivars arose from multi-allelic mutations of the *MYBA1* and *MYBA2* genes (Kobayashi et al., 2004; Lijavetzky et al., 2006; Walker et al., 2007), which control a rate-limiting step in anthocyanin synthesis. In muscadine grape, the *MybA1* and *MYBCS1* genes are upregulated in the skins of berries at veraison and maturity in red cultivars, and the transcription of *VrMybA1* and *VrMYBCS1* in the berry skin positively correlates with anthocyanin accumulation. The results of another muscadine berry study revealed that the maximum accumulation of phenolics occurs between 72 and 109 days after flowering and decreases during veraison, while total antioxidant activity, which is dependent on phenolics concentration, reached the highest in ripening berries (Mbele et al., 2008).

In the present study, we aimed to decipher the genetics and developmental differences between two cultivars of muscadine grape using morphological characterization as well as physiological and transcriptional analysis. The transcriptional profiles of grape berry were characterized at the last three stages of ripening in the muscadine grape cultivars “Carlos” and “Noble,” We identified new genes and molecular networks that are involved in the transition of berry ripening in both cultivars as well as in just one cultivar. We found stage-specific gene regulation for berry quality traits in “Carlos” and “Noble,” including genes in response to karrikin, inositol phosphoceramide synthase genes, and kinase receptor signaling genes. Those genes were discovered for the first time in grape berry ripening. In addition, we found new transcriptional factors in plant phase transition during flowering and fruit development such as BBM, PIF3, and HAC1. We further identified cultivar-specific genes in either “Carlos” or “Noble.” For example, wax and cuticle genes were enriched in the early stage of ripening in “Carlos.” In “Noble,” DEGs were enriched in response to hormone and hormone stimulus, including ABA and GA signaling. Furthermore, we discovered that developmentally and environmentally regulated pathways and cross-talk with other signaling pathways have a profound impact on muscadine grape berry development. Moreover, the new players characterized in this study give insight into the distinctive features of muscadine grape. The data acquired in this study provide genetic resources for grape breeding.

## Materials and methods

### Grape sample preparation

Two cultivars of muscadine grape, “Carlos,” with medium bronze fruit, and “Noble,” with dark purple fruit, were harvested separately at three berry developmental stages (bunch closure, veraison, and maturity) from a vineyard located at the University of Florida Plant Science Research and Education Unit (Latitude 29.40 N, Longitude 82.17 W, Altitude 21 m) in Citra, Florida, United States. After harvest, the berries were transported in a cooler to the postharvest laboratory at the University of Florida in Gainesville, FL. The samples were harvested at the same calendar day for both cultivars in the morning. The samples at the three stages were harvested on 16th June, 22nd July, and 6th August 2020, respectively. The berries were placed in sealed bags and stored in a freezer at −30°C until analysis. Four replicates of 20 berries of each ripening stage were used for various chemical and real-time PCR (RT-PCR) analyses.

### Titratable acidity and total soluble solids

The total soluble solids (TSS) and titratable acidity (TA) were only measured in berries harvested at the mature stage since TSS is an important feature of fruit quality. Twenty berries without seeds were homogenized for each replication and centrifuged for 20 min at 12,000 rpm and 4°C. The supernatant was filtered through cheesecloth. The TSS and TA measurements were carried out on the juice samples. The TSS was measured using an automatic temperature-compensated refractometer (Reichert R^2^i300; NY, United States). The TA was determined using an automatic titrator (Metrohm 848 Titrino plus, Herisau, Switzerland). The juice samples were titrated to pH 8.2 with 0.1 M NaOH after recording the initial pH and expressed as a percentage of citric acid, malic acid, and tartaric acid equivalents in juice.

### Total antioxidant capacity

The antioxidant capacity of the juice was determined by the ferric reducing antioxidant power (FRAP) assay (Benzie and Strain, 1996) with slight modification. The FRAP reagent was made with 300 mmol/l acetate buffer (pH 3.6) and 10 mmol 2,4,6-Tris(2-pyridyl)-1,3,5-triazine (TPTZ) in a 40 mmol/l HCl solution and 20 mmol/l FeCl_3_ in a 10:1:1 ratio. In a yellow light environment, 20 μl of a juice sample or 20 μl of the different concentrations of Trolox solutions (0 to 500 μmol/l) were mixed with 980 μl of FRAP reagent. The samples and the standards were read at 595 nm with a microplate reader. FRAP values were calculated from a standard curve of Trolox and expressed as μmol of Trolox equivalent per gram of fresh weight (μmol TEAC/g FW).

### Total phenolic compounds

Total phenolic content (TPC) was determined using the modified colorimetric Folin–Ciocalteu method (Singleton and Rossi, 1965) with slight modification. A mixture of berry peel and flesh (5 g) was extracted with a mixture of 30 ml formic acid, 600 ml methanol, and 370 ml water and kept in a refrigerator overnight at 4°C. After centrifuging for 20 min at 12,000× g rpm and 4°C, a 300-μl aliquot of supernatant was mixed with 300 μl Folin–Ciocalteu reagent and 300 μl sodium carbonate. The mixture was left in a dark room for 60 min at room temperature before measuring the absorbance at 765 nm with a microplate reader. The same procedure was applied for standard solutions of different concentrations (0–200 mg/l) of gallic acid. The phenolic content was expressed as mg of gallic acid equivalents per gram of fresh weight (mg GAE/g FW).

### Total anthocyanin content

Total anthocyanin content (TAC) was measured according to the pH-differential method (Giusti and Wrolstad, 2001) using two buffer systems: 0.4 M sodium acetate (pH = 4.5) and 0.25 M potassium chloride (pH = 1). The extract supernatant (600 μl) was prepared above (TPC measurement) and was mixed with 2.4 ml of the sodium acetate and potassium chloride buffers. The absorbance of the samples was measured at 510 nm and 700 nm using a microplate reader, and each sample was measured in triplicate (*n* = 3) at room temperature (∼22°C). Total anthocyanin content (mg/100 g FW) was measured as equivalents of cyanidin-3-glucoside (C-3-G) as calculated by the following equation:


$$A={\left(A_{pH1.0}-A_{pH4.5}\right)}_{510nm}-{\left(A_{pH1.0}-A_{pH4.5}\right)}_{700nm}$$

C−3−G(mg/L)=(A×MW×DF×1,000)/L×ε

Where A is absorbance, MW is molecular weight and DF is dilution factor, L denotes pathlength, ε denotes molar extinction coefficient, and 1,000 is the conversion factor from gram to milligram.

### RNA extraction and RNA sequencing

RNA was extracted from two different cultivars of muscadine grapes, “Carlos” and “Noble.” Grape berries from the last three developmental stages, namely bunch closure, veraison, and maturity (harvest), were collected from the research station (Citra) at University of Florida. Total RNA was isolated from each of the developmental stages using TRIZOL (Ambion, Life Technologies), followed by DNase treatment (Turbo DNA free, Thermo Fisher). RNA-seq libraries were constructed at the ICBR Gene Expression and Genotyping Core Lab using NEBNext® Ultra™ Directional RNA Library Prep Kit for Illumina (New England Biolabs, Ipswich, MA, United States) following the manufacturer’s recommendations. A total of 1,000 ng samples of high-quality total RNA (RNA Integrity Number, RIN ≥ 7) was used and followed by RNA library preparation with NEBNext Ultra II Directional Lib Prep (New England Biolabs, Ipswich, MA, United States, catalog #E7760) according to the manufacturer’s user guide. In general, RNA was fragmented and followed by first strand cDNA synthesis using reverse transcriptase and oligo dT primers. Synthesis of ds cDNA was performed using the 2nd strand master mix provided in the kit, followed by end-repair and adaptor ligation. The library was enriched by 8 cycles of amplification, and purified by Agencourt AMPure beads (Beckman Coulter, catalog # A63881). Finally, the individual libraries were pooled using equimolar amounts and sequenced by Illumina NovaSeq 2,150 cycles run for a total of 0.5 lanes (Illumina Inc., CA, United States).

### RNA-sequencing data analysis

The quality of the RNA-Seq sequence data was evaluated using FastQC (FASQC)^1^ prior to further downstream analysis. Low-quality sequences were trimmed, and poor-quality reads were removed using Trimmomatic (Bolger et al., 2014). The bowtie Aligner (Dobin et al., 2013) was used to map high-quality paired-end reads to the genome of GCF_000695525.1 (hg19).^2^ Gene expression was obtained using RSEM^5^. The estimated read counts were used as input for edgeR^6^ to perform differential expression (DE) analysis. The exact test model was used to identify DE genes. The thresholds were set at an FDR of 0.05 and a fold change of greater than 2.

GO term analyses were performed at http://plantregmap.gao-lab.org/go_result.php using *Vitis vinifera* as a reference genome. The gene regulatory network and KEGG analyses were conducted through STRING.^3^ The transcription factor identification analysis was through Plant Transcriptional Regulatory Map.^4^

### Real-time PCR analysis

RT-PCR was performed to validate the expression for 15 genes observed as differentially expressed in the transcriptomic data sets. First-strand cDNA synthesis with 1 μg of total RNA isolated above was performed using a reverse transcription kit (Applied Biosystems). For quantitative RT-PCR, primers were designed using Primer Quest available from Integrated DNA Technologies (IDT). The primers for each selected DEG are listed in Table 1. Real-time PCR reactions were performed in an Applied Biosystems qPCR (Thermo Fisher). Each reaction was 20 μl. Each gene was amplified in triplicate reactions with thermocycler conditions of 95°C for 10 min and 45 cycles for 95°C for 30 s and 60°C for 30 s. Relative expression of each gene was normalized to the Ct value of actin (internal control), and relative expression was calculated using 2^−∆∆Ct^. All the values shown are mean ± SE.

**Table 1:** TA and TSS of muscadine grape (cultivars “Carlos” and “Noble”) harvested at maturity stage.

Cultivar	Citric acid (g 100 g^−1^)		Malic acid (g 100 g^−1^)		Tartaric acid (g 100 g^−1^)		TSS (°Brix)	
Noble	0.63	a	0.66	a	0.73	a	15.6	a
Carlos	0.73	a	0.76	a	0.85	a	12.9	b

TSS, Total soluble sugars.

## Results and discussion

### Morphological and physiological characterization of muscadine grape during fruit ripening

**Figure 1:**
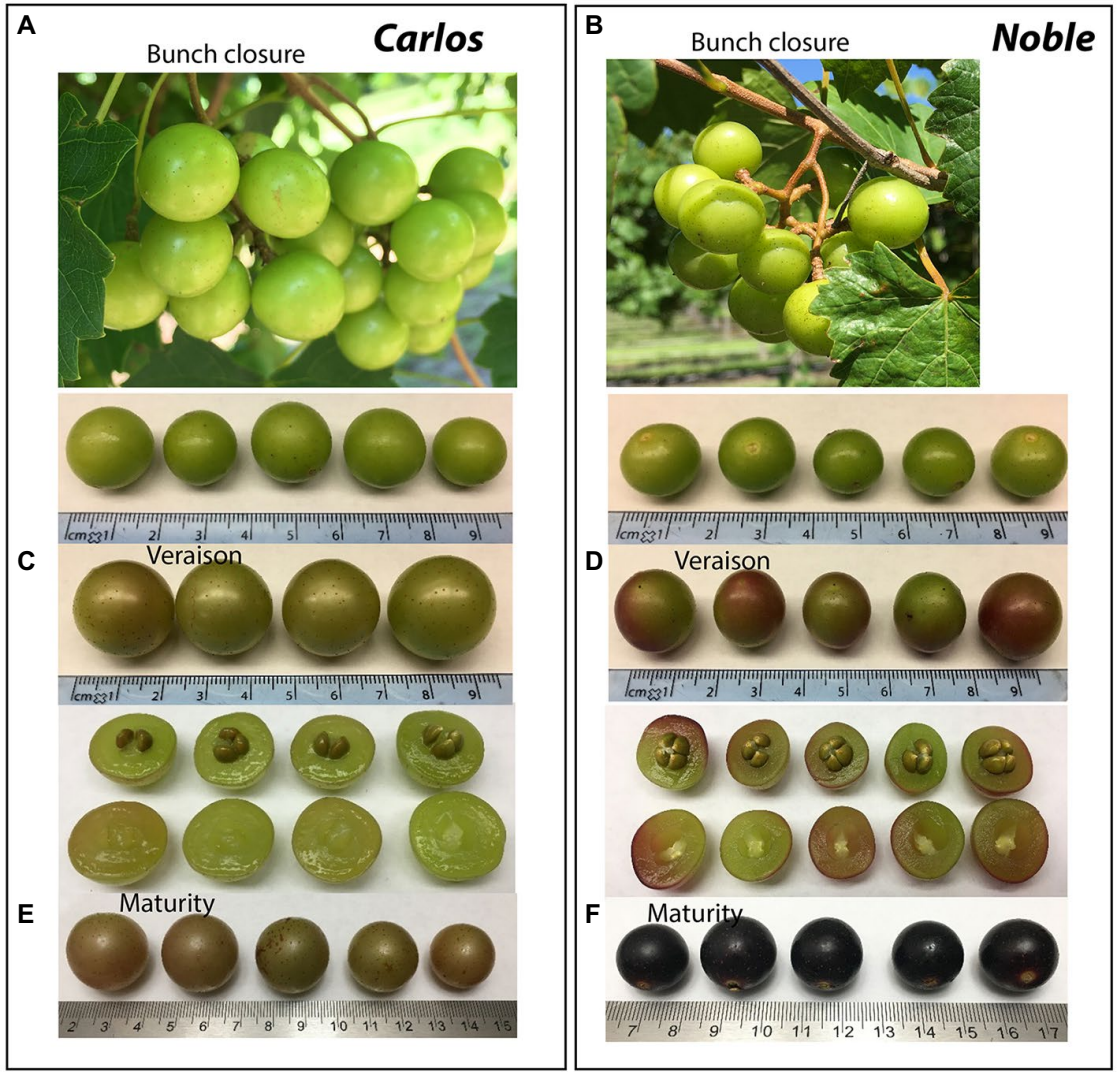
Representative images of muscadine grape genotypes during ripening. The photographs show the muscadine grape berries of “Carlos” on the left **(A,B,C)** and “Noble” on the right **(D,E,F)** at bunch closure (E-L 32), veraison (E-L 35), and maturity stage (E-L 38), respectively.

The cultivars “Noble” and “Carlos” are the industry standards for red and white muscadine wine production, respectively (Figures 1A,B). Both cultivars are highly vigorous and productive, produce a medium-small-sized fruit, ripen mid-season, and are self-fertile. Starting from the bunch closure stage, the berries of both cultivars are green in color with light brown scar spots (Figures 1A,B). At the veraison stage, “Carlos” berries remain green, while “Noble” berries are half green and half purple (Figures 1C,D). At the maturity stage, “Carlos” produces bronze berries with a dry stem scar and “Noble” produces purple berries with a wet stem scar (Figures 1E,F). Berry ripening is classified into seven developmental stages according to a modified Eichhorn-Lorenz (E-L) system, including bunch closure (S5/E-L 32), veraison (S6/E-L 35), and maturity (S7/E-L 38; Coombe, 1995). The transition from veraison to ripeness is the final stage of grape development. Bunch closure stage was identified as the beginning of berries touching. At this stage, the berries were still green and hard, but they started to soften and accumulate sugar. At veraison stage, the berries continued softening and accumulating sugar, and they began to color and enlarge. At maturity stage, they are soft and sweet enough for consuming and fully colored (Coombe, 1995; Carbonell-Bejerano et al., 2016; Canaguier et al., 2017; Ghaffari et al., 2020).

### Chemical composition in “Carlos” and “Noble” berries

Titratable acidity (TA) and total soluble solids (TSS) were only measured at maturity stage (Table 1). There were no significant differences in the percentage of citric acid, malic acid, and tartaric acid between the two cultivars. However, the TSS, represented as °Brix value, was significantly higher, by 21%, in “Noble” than in “Carlos.” The TSS/TA ratio was calculated as a sweet–sour taste sensation indicator. In “Noble,” the TSS and TA measurements of 15.6 (°Brix) and 0.73 (g 100 g^−1^ tartaric acid equivalent), respectively, yielded a °Brix/acid ratio of 21.4. The °Brix/acid ratio of “Carlos” was 15.2. According to Nelson et al. (1973), consumers prefer muscadine grape cultivars with a higher °Brix/acid ratio.

**Figure 2:**
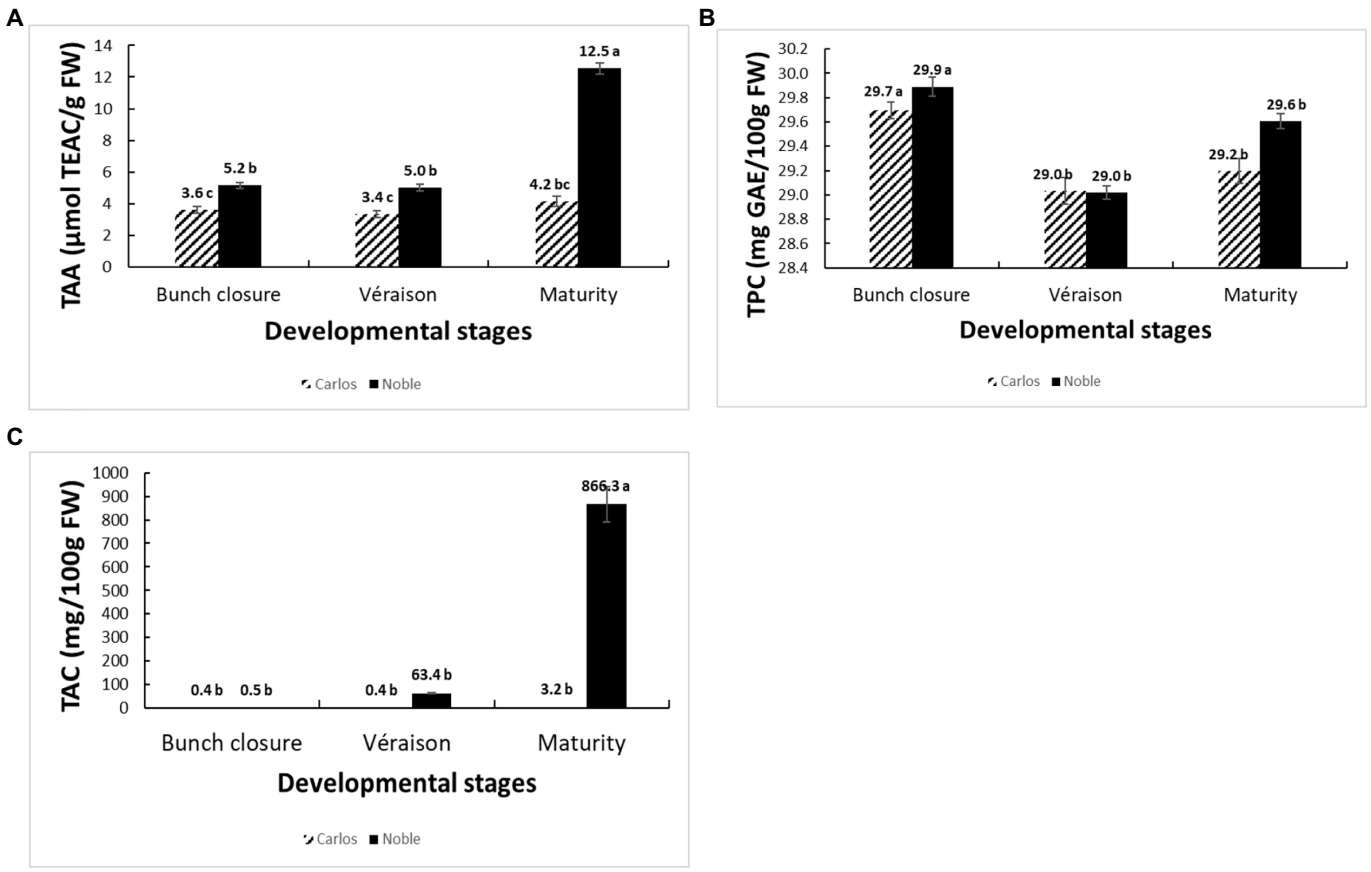
Physiological and biochemical changes of muscadine grapes cv. “Carlos” and “Noble” during grape berry ripening. The developmental stages include bunch closure, veraison, and maturity. **(A)** Total antioxidant activity (TAA) was measured by the ferric reducing antioxidant power (FRAP) assay. **(B)** Total phenolic content (TPC) was measured by the modified colorimetric Folin–Ciocalteu method with slight modification. **(C)** Total anthocyanin content (TAC) was measured by the pH-differential method. The experiments were carried out in four biological replicates, and each replicate was repeated three times. The different lowercase letters represented significant differences among the developmental stages of an individual cultivar, according to the Glimmix procedure in SAS 9.4 (*p* > 0.05).

The total antioxidant activity (TAA) was measured from the juice of berries without seeds (flesh and skin only), expressed as μmol of Trolox equivalent per gram of fresh weight (μmol TEAC/g FW). On the basis of fresh weight, the total antioxidant activity was 12.5 μmol of TEAC/g FW in “Noble” at maturity, which was significantly higher than the other two stages as well as all three stages of “Carlos” (Figure 2A). The TAA of “Carlos” showed no significant change at the three harvested stages. The total phenolic content (TPC) and total anthocyanin content (TAC) were measured from the extract of berry flesh and skin mixture (no seeds), which were expressed as mg of gallic acid equivalents per gram of fresh weight (mg GAE/g FW) and mg per 100 g fresh weight (mg/100 g FW), respectively. The TPC was highest at bunch closure and significantly lower at veraison in both cultivars (Figure 2B). At maturity, the TPC in “Carlos” did not change, but it did increase significantly in “Noble.” The TAC differed greatly between the green and red cultivars. In the green “Carlos” fruit, there was almost no anthocyanin production at all stages, while “Noble” showed a small increase at veraison and a dramatic increase by maturity (Figure 2C). This was consistent with visual observation of the maturing berries, as shown in Figure 1. Both cultivars displayed green color at bunch closure. At veraison, “Carlos” remained green, but “Noble” started turning purple. At maturity, “Carlos” turned to a bronze color and “Noble” turned completely purple.

We found distinctive berry characteristics at each stage for each cultivar. The total antioxidant activity was significantly higher in “Noble” than in “Carlos.” “Noble” at maturity displayed the highest antioxidant activity according to the FRAP assay, which might be due to the high anthocyanin accumulation. The total phenolic content declined from bunch closure to veraison and did not significantly increase by maturity, which was similar to results in the muscadine cultivar “Late Fry” (Darwish et al., 2021). Moreover, “Noble” started to accumulate anthocyanin at veraison, but most accumulated at maturity, while “Carlos” only produced a small amount of anthocyanin at maturity. Many factors influence the amount of phenolics and antioxidants, including genotypic differences, environmental conditions, and, most critically, developmental phases (Darwish et al., 2021). Since differences among developmental phases are reflections of changes in gene expression, we utilized RNA-sequencing to further study the physiological and molecular changes that occur during berry development.

### Common and specific switch gene during muscadine fruit ripening

**Figure 3:**
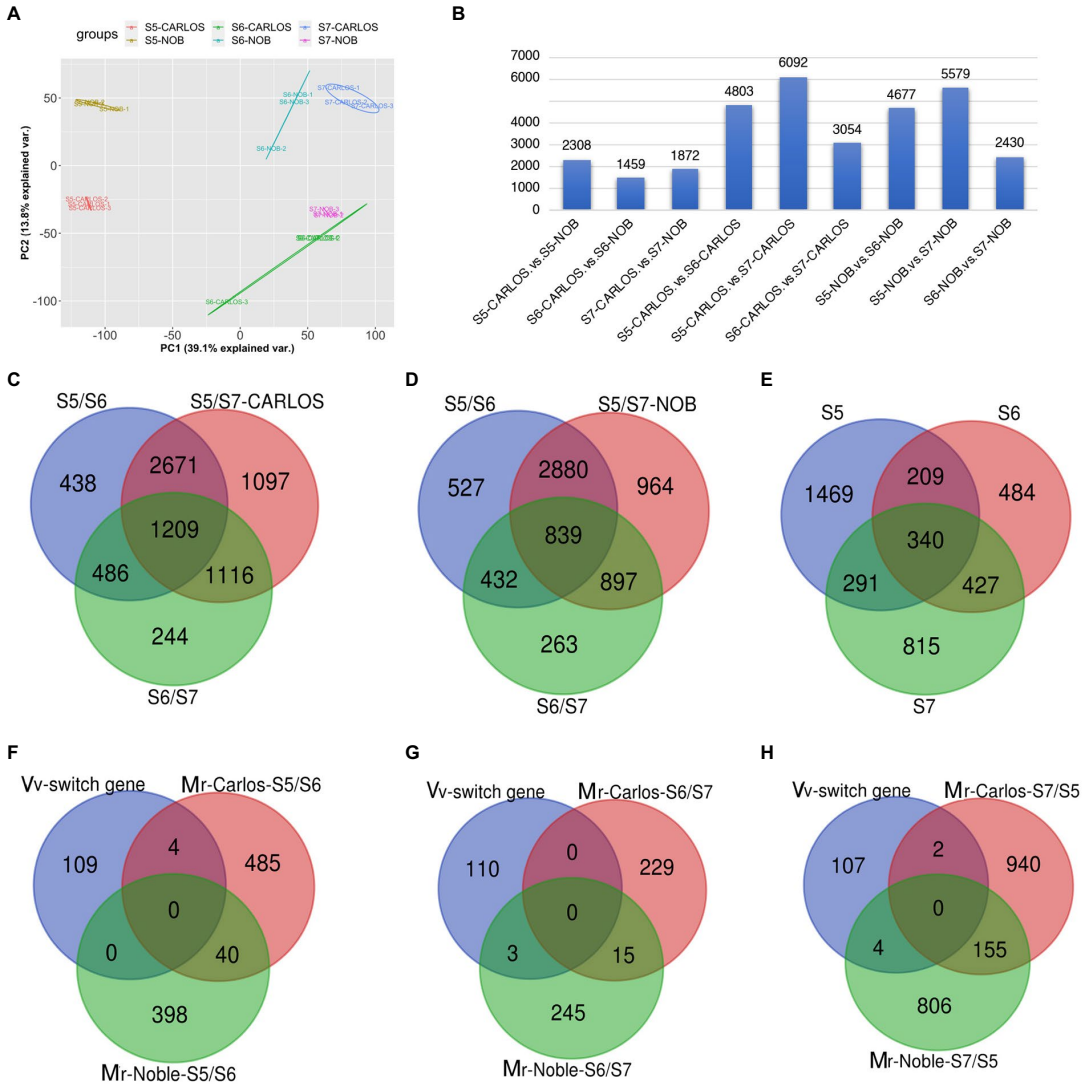
Gene expression patterns in the grape samples. **(A)** Principal component analysis (PCA) of transcriptome data from three stages of fruit ripening in two cultivars. Color code is shown. Each dot represents one sample. Color-coded lines and cycles show the replicates. **(B)** Total number of differentially expressed genes (DEGs) between cultivars and between two stages in one cultivar. Blue bar represents the number of DEGs. **(C,D)** Venn diagram illustrating the overlap of DEGs among E-L 32, E-L 35, and E-L 38 in “Carlos,” **(C)**; and in “Noble,” **(D)**. **(E)** Total number of overlapping DEGs in both cultivars. **(F,H)** Stage-specific genes among the common switch genes in *V. vinifera* (Vv) and *M. rotundifolia* (Mr) *“*Carols” and “Noble” in (E-L 32) **(F)**, (E-L 35) **(G)**, and (E-L 38) **(H)**. Bunch closure (S5/E-L 32), veraison (S6/E-L 35), and maturity (S7/E-L 38).

To investigate the transcriptional changes that occur during grape berry ripening, we performed RNA-sequencing of two cultivars, “Carlos” and “Noble,” at the last three stages: bunch closure (E-L 32), veraison (E-L 35), and maturity (E-L 38). Three biological replicates of muscadine grape samples were sequenced for each cultivar. Principal Component Analysis (PCA) of the three replicates displayed strong correlations for the different stages of both cultivars, suggesting that the experiments had good reproducibility and reliability (Figure 3A).

A summary of RNA-seq data from the two genotypes and three developmental stages of muscadine grape is shown in Figure 3B. There were 5,639 differentially expressed genes (DEGs) between the two cultivars across the three fruit ripening stages. There were 2,308 DEGs that were differentially expressed at bunch closure (E-L 32); 1,459 genes were at veraison, and 1,872 genes were at maturity (Figure 3B). The bunching grape (*Vitis vinifera*) genome data predict 30,434 genes (Jaillon et al., 2007). That means that bet about 20% (5,639/30,434) of the genes are differentially expressed between the “Carlos” and “Noble” cultivars across the three fruit ripening stages. We sought to identify the common and veraison- and maturity-specific genes (Palumbo et al., 2014) during muscadine fruit ripening. In “Carlos,” there were 4,803 DEGs between E-L 35 and E-L 32, 3,054 DEGs between E-L 35 and E-L 38, and 6,092 DEGs between E-L 38 and E-L 32 (Figure 3B). Similarly, in “Noble,” there were 4,677 DEGs between E-L 35 and E-L 32, 2,430 DEGs between E-L 38 and E-L 35, and 5,579 DEGs between E-L 38 and E-L 32 (Figure 3B). These patterns are consistent and reveal a large change in expressed genes at maturity in both cultivars compared to the other two stages, suggesting that this period is the major transition during berry ripening (Figure 3B).

### Stage-specific gene regulation for berry quality traits in “Carlos” and “Noble”

Comparison of the overlapping DEGs across the three stages revealed a total of 1,209 genes in “Carlos” and 839 in “Noble” that changed levels throughout ripening (Figures 3C,D). The majority of overlapping genes were involved in carbohydrate metabolism and cell wall organization and biosynthesis (Supplementary Figures 1A–C). Interestingly, xyloglucan biosynthesis and xyloglucosyl transferase genes are among these overlapping genes. These genes catalyze the cleavage of a beta-bond in the backbone of a xyloglucan and transfer the xyloglucanyl segment to xyloglucan during cell wall modification (Han et al., 2015). Our results suggested that these enzymes play a role in the remodeling and restructuring of the cell wall during berry growth and softening.

We further identified 438 E-L 32-specific, 244 E-L 35-specific, and 1,097 E-L 38-specific genes in “Carlos” (Figure 3C). The number of genes enriched in gene ontology (GO) terms related to response to stress stimulus increased from E-L 32 to E-L 38 (Supplementary Table 1). We assessed the function of the E-L 35-specific transcripts and found genes enriched in cellular process, homeostasis, and multicellular organismal development. These data imply that developmental transition occurs to modulate quantitative traits such as size, biomass, shape, and color (Supplementary Figures 1A–C). Additionally, we found 58 transcription factors expressed at this stage, including genes in the AP2/ERF, GATA, and bZIP transcription factor families (Supplementary Table 1). These transcription factors may play an important role in phase transitions. Among the 1,097 E-L 38-specific genes, we found genes enriched in cellular process, signaling proteins, cell cycle, and cellular transport (Supplementary Table 1). Pigment metabolic process genes are also highly expressed in this stage. Interestingly, we found genes in response to karrikin, including *VvKAI2* (VIT_07s0129g00530, a homolog of *KAI2*, *AT4G37470*) and a MYB gene (VIT_17s0000g07510, *AT1G70000*). Karrikin, a family of compounds produced by wildfires that can stimulate seeds germination (Flematti et al., 2013), might be a signaling molecule during berry ripening.

**Figure 4:**
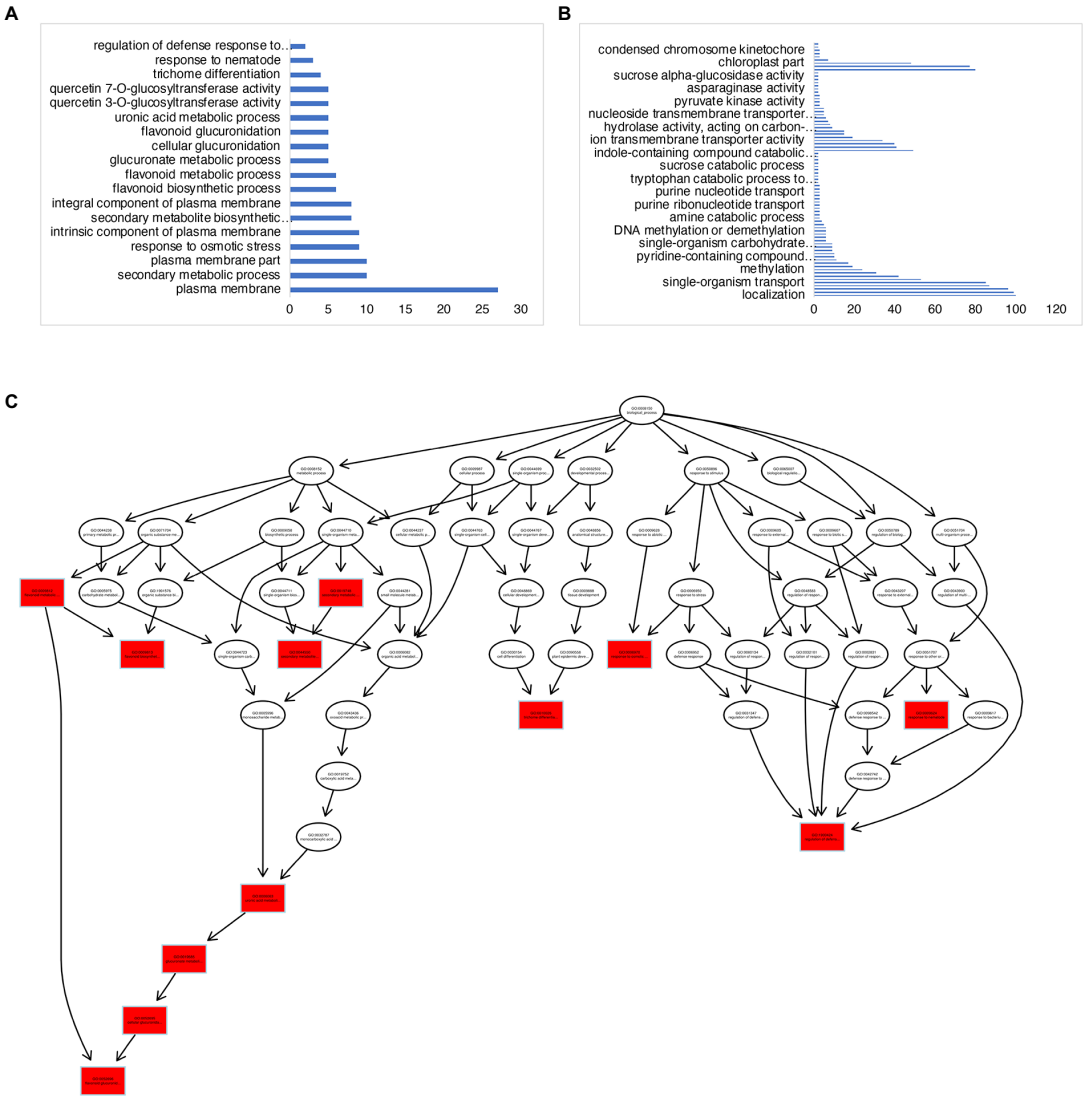
GO-term analysis on the E-L 35- and E-L 38-specific genes in “Noble.” **(A,C)** GO terms analysis of the E-L 35-specific genes showing enrichment for a GO term were in flavonoid biosynthetic and metabolic process, quercetin and glucuronate biosynthesis, and metabolic process. **(B)** Genes expressed at maturity (E-L 38) that were involved in methylation and epigenetic modification.

There were a total of 839 DEGs that overlapped across all stages of “Noble” berries (Figure 3D; Supplementary Table 2). In addition to cell wall-associated genes and xyloglucan, which are consistent with “Carlos,” we found genes in response to heat and light, suggesting that warmer temperature and light intensity may promote ripening (Supplementary Table 2). We next identified 527 E-L 32-specific, 263 E-L 35-specific, and 964 E-L 38-specific genes in this cultivar (Figure 3D). Inositol phosphoceramide synthase genes and kinase receptor signaling genes were activated in this stage (Supplementary Figure 2), suggesting that signaling molecules such as inositol might be involved in gene regulation. Interestingly, the majority of the E-L 35-specific genes showing enrichment for a GO term were in flavonoid biosynthetic and metabolic process, quercetin and glucuronate biosynthesis, and metabolic process, suggesting that pigment, odor, flavor, and textures developed in this stage (Figures 4A,C).

We next examined the transcriptional regulations among these genes through the TF Enrichment Tool,^5^ which identifies transcription factors from the literature and ChIP-seq data or through inference by combining TF binding motif and regulatory element sequences.^6^ This analysis revealed 43 transcription factors among the E-L 35-specific genes, including two *Squamosa Promoter-Binding-Like* (*SPL*) genes (*VIT_12s0028g03350* and *VIT_07s0005g02260*), a MADS box floral homeotic gene orthologous to *AGAMOUS* (*VIT_10s0003g02070*), and a floral homeotic gene orthologous *PISTILLATA*, (*VIT_18s0001g01760*; Supplementary Table 2). *SPL*, *AGAMOUS* and *PISTILLATA* genes encode plant-specific transcription factors that play important roles in plant phase transition, flowering, and fruit development (Honma and Goto, 2001). Furthermore, the *BBM* homolog *VIT_204s0023g00960*, a meristem development gene, was also expressed in this stage. This suggests that growth regulators are key players during tissue transition and maturation.

Among the 964 genes expressed at maturity (E-L 38), 70 (7%) genes were related to methylation, including methylation-dependent chromatin silencing, methyltransferase activity, and epigenetic modification (Figure 4B; Supplementary Table 2). These data are consistent with previous research on “switch genes” that are present at the immature-to-mature transition during ripening and with the high degree of DNA methylation, small interfering RNA (siRNA), and epigenetic modification that are activated during developmental transitions (Palumbo et al., 2014). The E-L 38-specific genes included 78 transcription factors. We found four *PIF3* homologous genes, *VIT_14s0060g00260*, *HAC1* (a transcriptional activator, ortholog of HY5), *VIT_04s0008g05210*, and *MYB15*, *VIT_07s0005g02570,* suggesting that light signaling may modulate grape berry maturation.

### Gene expression variability between “Carlos” and “Noble” during berry ripening

The two cultivars shared a total of 340 DEGs among all three stages. These shared DEGs were enriched for GO terms associated with oxidation–reduction (redox) reaction in the molecular function category (Supplementary Table 3). There were 1,469, 484, and 815 DEGs specific to E-L 32, E-L 35, and E-L 38, respectively (Figure 3E). We compared these E-L 32-, E-L 35-, and E-L 38-specific DEGs between the “Carlos” and “Noble” cultivars (Figures 3F–H). We also included the 110 switch genes that were identified in previous research as master regulators during the transition from immature to mature growth in grapevine (*V. vinifera*; Palumbo et al., 2014). At E-L 32, there were 398 DEGs unique to “Carlos” and 489 DEGs unique to “Noble,” and 40 that were shared (Figure 3F). This means that only a small number of DEGs overlap between those two cultivars at the early ripening stage (Figure 3F). Among the 398 DEGs in “Carlos,” one-third (132 genes) were enriched in stress response GO terms such as abiotic, heat, or radiation stress. Interestingly, DEGs were enriched in wax and cuticle biosynthesis and metabolic process, suggesting that wax and cuticle genes were required in the early stage of ripening in “Carlos” (Supplementary Figure 3; Supplementary Table 4). In “Noble,” DEGs were enriched in response to hormone and hormone stimulus (Supplementary Table 4), including ABA and GA signaling.

A total of four switch genes overlapped with E-L 32/E-L 35 specific genes in “Carlos,” including *VIT_14s0060g00270*, a DYW-deaminase domain-containing protein; *VIT_05s0062g00980* and *VIT_05s0062g00990*, aldo_ket_red domain-containing proteins; and *VIT_12s0028g03580*, an uncharacterized protein (Figure 3F). We identified 229 DEGs in “Carlos” and 248 DEGs in “Noble” at E-L 35 (Figure 3G). The shared 15 DEGs are all involved in response to stresses (Figure 3G), particularly salt and osmotic stress. There was significant enrichment of GO terms in cellular homeostasis, regulation of flower development, and sulfur compound metabolic process in the 229 DEGs between E-L 35/E-L 38 in “Carlos.” In contrast, glucuronate metabolic process, cellular glucuronidation, and flavonoid glucuronidation were enriched among the 248 DEGs of “Noble” (Supplementary Table 4). We next examined the 154 DEGs shared in E-L 38 that were involved in pyridine nucleotide metabolic process (Supplementary Table 4). Pyridine nucleotides such as nicotinamide adenine dinucleotide (NAD) are universal cornerstones of plant metabolism which are ubiquitous electron carriers modulating energy homeostasis through the transport of electrons within reduction–oxidation (redox) processes. NAD metabolism and signaling are very dynamic during fruit development, and its differential regulation is certainly critical to linking central metabolism with berry maturation and ripening. We found three overlapping switch genes among the E-L 35/E-L 38-specific DEGs, including *VIT_04s0023g02510*, a glycos_transf_1 domain-containing protein; *VIT_01s0011g03670*, an Aspergillus nuclease S (1) encoding an Endonuclease 4; and *VIT_14s0066g01710*, a PMR5N domain-containing protein, a protein trichome birefringence-like 9 related genes (Figure 3G; Supplementary Table 4). In mature “Carlos” berries, there were 942 DEGs not present in “Noble” berries, including 36 genes in fruit or seed development and 4 genes in floral organ abscission (Figure 3H). These genes regulate shedding of floral organ, fruit maturation, and seed development. The genes MYB60 and YABBY are involved in fruit shape and size. The genes *MYB60* and *YABBY* are involved in fruit shape and size. In grapevine, the *VvMYB60* transcription factor is involved in regulating stomatal activity and is differentially expressed in response to ABA and osmotic stress (Galbiati et al., 2011). Another gene, *VvDAM* (*VIT_00s0313g00070*), is involved in fruit development in grapevine (Shangguan et al., 2020) and is repressed by the floral homeotic genes *AP1* and *SEP3* in emerging floral meristems (Sridhar et al., 2006) but upregulated by *HUA2* (Chen and Meyerowitz, 1999; Doyle et al., 2005), *AGAMOUS-like 6* (Boss et al., 2002), *MADS box* (Boss et al., 2002), and *IDD7* genes. In mature “Noble” berries, there were 810 unique DEGs, including those enriched in sucrose metabolic process and nitrogen compound transport. Among the genes predicted as switch genes in grapevine, six were DEGs in the mature muscadine berries. Two switch genes were unique to mature “Carlos” berries. The E-L 38-specific genes are *VIT_08s0040g02880*, an uncharacterized protein, and *VIT_12s0028g03580*, an L-type lectin-domain containing receptor kinase-related gene. Four switch genes were specific to mature “Noble” berries including *VIT_16s0098g01150*, a small Auxin-up RNA-related gene; *VIT_13s0019g00*460, a DDE TNP4 domain-containing protein; *VIT_17s0000g08770*, a cysteine-rich Receptor-like Protein Kinase 2; and *VIT_13s0019g03910*, an uncharacterized protein.

### Developmental and environmental co-expression networks control muscadine grape berry maturation

**Figure 5:**
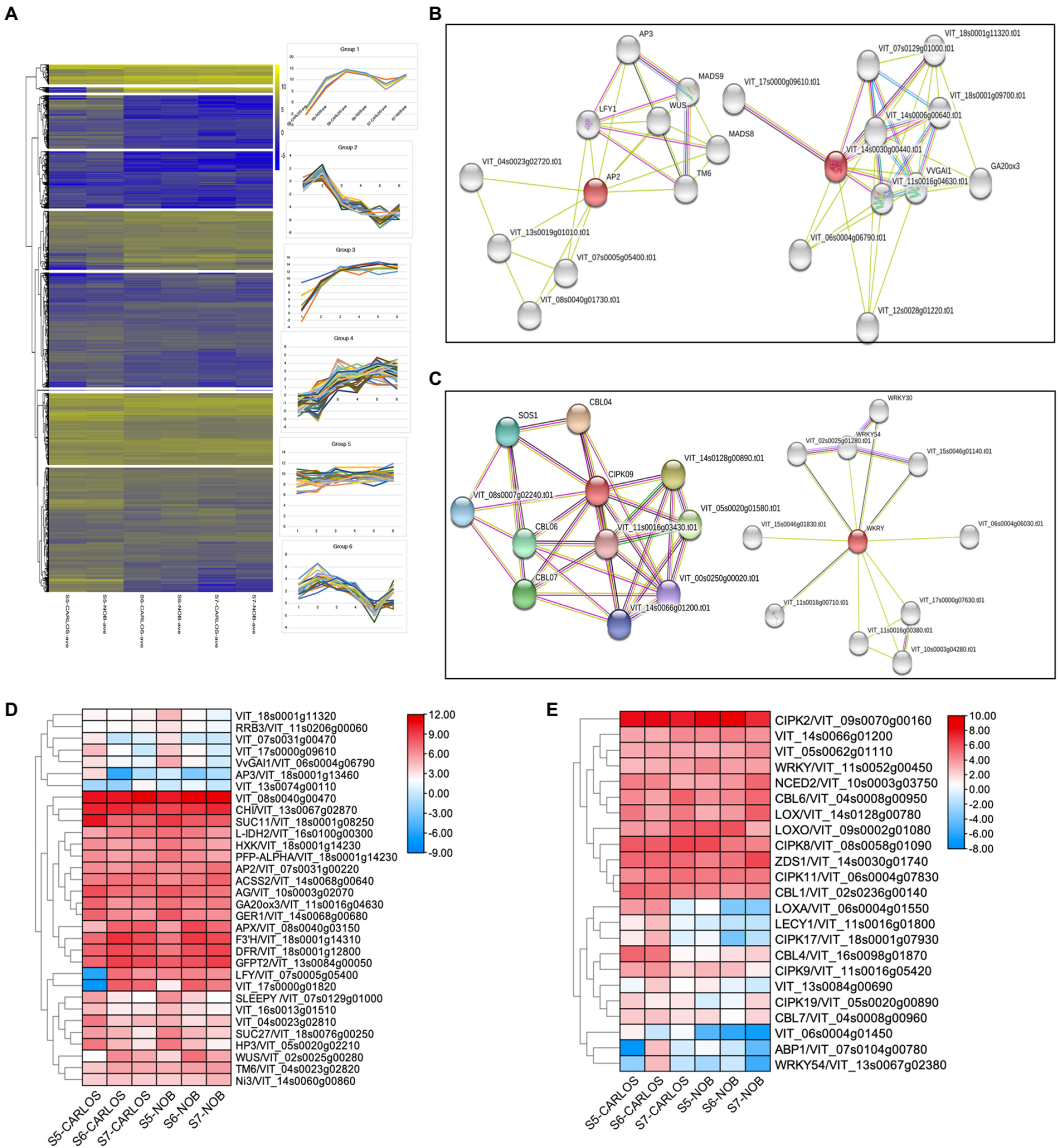
Gene regulatory networks in berry ripening. **(A)** Cluster analysis revealed seven expression patterns in this experiment. **(B)** Clusters from functional network analysis of the developmental hub genes of *AP2* on the left and *GA20ox3* on the right. **(C)** Functional network analysis of the environmental hub genes *CIPK09* on the left and *WRKY* on the right. **(D)** Heatmap showing the expression patterns of developmental regulators. **(E)** Heatmap showing the expression patterns of environmental regulators.

To obtain gene expression profiles for different berry ripening stages of two muscadine cultivars, RNA-seq data were grouped into nine different clusters using *k*-mean analysis (Figure 5A; Supplementary Table 5). The cluster diagram was based on the expression patterns of genes at three berry ripening stages. For example, Group 1 included 516 genes (Supplementary Table 5) that all showed an increasing trend from E-L 32 to E-L 38 in both “Carlos” and “Noble;” while the genes in Group 2, on the other hand, showed a decreasing trend from E-L 32 to E-L 38 (Figure 5A).

To further identify key genes, genes associated with regulatory networks, as well as potential protein interaction networks, we located DEGs within their associated networks through the STITCH Database (Figures 5B–E).^7^ We found master developmental regulators and identified co-expressed genes throughout berry maturation, which included the transcription factors *VvAP2* (*VIT_07s0031g00220*), *VvLFY* (*VIT_07s0005g05400*), *VvWUS* (*VIT_02s0005g00280*), and *VvAG* (*VIT_10s0003g02070*) that play roles in the transition from meristem to organ development (Figure 5B). Previous research in grapevine ripening has demonstrated that AP2 and other ERF genes are the main factors in determining floral and fruit maturation (Licausi et al., 2010). These genes were all expressed during ripening in berry skin and flesh to enhance maturation.

Other genes up-regulated during grape berry ripening are chalcone flavanone isomerase (*VrCHI*, *VIT_13s0067g02870*), *flavanone 3-hydroxylase* (*F3H, VIT_18s0001g14310*), and *dihydroflavonol 4-reductase* (*DFR, VIT_18s0001g22800*). These genes are important in the biosynthesis of antioxidant flavonoid pigments (Figure 5D). These genes showed preferred expression in the maturation stage of berry ripening. Interestingly, we noticed a considerable number of sugar-signaling genes involved in berry ripening. This is consistent with previous studies showing that hexokinase (*HXK*, *VIT_18s0001g14230*) activity is reduced after the onset of ripening (Wang et al., 2014). Similarly, the expression levels of genes that co-express with *HXK*, namely *VvSUC27* (*VIT_18s0076g00250*), *VvSUC11* (*VIT_18s0001g08250*), and isocitrate dehydrogenase gene (*VvIDH1*, *VIT_16s0100g00300*), were drastically reduced (Figure 5D). There was also considerable spatial variation in hormone-associated genes, such as *GA20ox3* (*VIT_11s0016g04630*) and *SLEEPY* (*SLY1*, *VIT_07s0129g01000*), key gibberellin (GA) biosynthesis, and signaling genes, respectively (Figures 5B,D). GAs are well-known for their roles in the control of bud dormancy and fruit and seed development in grapevine (Zheng et al., 2015). Interestingly, both GA20ox3 and SLY1 showed predominant expression at the onset of berry ripening and gradually reduced with the progression of ripening (Figure 5B). Our results suggest that GAs are involved in berry enlargement and seeds maturation at the early stage of ripening, consistent with the application of GA for to manage fruit expansion and seed abortion in ripening grapevine (Zheng et al., 2015).

We next sought to identify hub genes and gene regulatory networks that are associated with stress responses. A large number of *calcineurin B-like proteins* (*CBL*s) and their target proteins, *CBL-interacting protein kinase* (*CIPK*s), were identified as differentially expressed at the onset and during ripening. Calcium (Ca) plays an essential role as a signaling messenger in intracellular communication in response to environmental cues (Chen et al., 2011). Both *CBL*s and *CIPK*s are key plant-specific players in Ca signaling networks in *Arabidopsis* and maize (Chen et al., 2011; Mao et al., 2016). We observed that *CBL6/VIT_04s0008g00950*, *CIPK2/VIT_09s0070g00160*, *CIPK8/VIT_08s0058g01090*, *CIPK11/VIT_06s0004g07830*, and *WRKY/VIT_11s0052g00450* were significantly enriched in E-L 38 berries (Figure 5E), which suggests these genes are key components in the regulation of stress signaling in muscadine grape. However, other CBL-CIPK signaling genes, such as *CBL4/VIT_16s0098g01870*, *CBL7/VIT_04s0008g00960*, *CIPK9/VIT_11s0016g05420*, *CIPK17/VIT_18s0001g07930*, and *CIPK19/VIT_05s0020g00890*, were downregulated and acted as negative regulators during ripening, which indicated that CBL-CIPK signaling showed complexity in response to different stresses during berry ripening (Figure 5E). Similarly, the abscisic acid biosynthesis gene, *NCED2/VIT_10s0003g03750*, was enriched at E-L 38. However, the expression patterns are different between “Carlos” and “Noble.” The jasmonic acid biosynthesis genes encoding lipoxygenase, *LoxA* and *LoxO*, showed distinct patterns during ripening (Yang et al., 2012). Taken together, our results revealed both the variety and the specificity of ABA and JA biosynthesis during grape berry ripening.

### Key pathways were activated during berry maturation

We next investigated the pathways active during berry maturation by conducting a Kyoto Encyclopedia of Genes and Genome (KEGG) pathway analysis on E-L 35- and E-L 38-specific genes in the two cultivars (Table 2). Genes in the sulfur metabolism pathways were enriched at stage E-L 35 in “Carlos.” In the table grape and wine industry, sulfur residue causes off-flavors and a negative value in grapes during fermentation (Kwasniewski et al., 2014). Therefore, it is important to investigate sulfur metabolism in muscadine grape and to understand the absorption and oxidation of SO_2_ in the berry.

**Table 2:** KEGG pathway analysis of genes from immature to maturity development.

Stages/cultivars	KEGG/Pfam pathway description	Observed gene count
E-L 35/E-L 32 “Carlos” genes	Metabolic pathways	48
	Sulfur metabolism	5
E-L 35/E-L 32 “Noble” genes	Fatty acid metabolism	10
	Peroxisome	9
	Fatty acid degradation	7
	Plant circadian rhythm	7
	Fatty acid elongation	4
	Folate biosynthesis	3
	Biosynthesis of unsaturated fatty acids	4
	Synthesis and degradation of ketone bodies	2
E-L 38/E-L 32 “Carlos” genes	Metabolic pathways	98
	Biosynthesis of amino acids	18
	Alanine, aspartate, and glutamate metabolism	8
E-L 38/E-L 32 “Noble” genes	Metabolic pathways	73
	Biosynthesis of secondary metabolites	51
	Glycolysis/gluconeogenesis	11
E-L 38/E-L 35 “Carlos” genes	Ribosomal Proteins L2, RNA binding domain	4
	KH domain	4
	Ribosomal Proteins L2, C-terminal domain	3
E-L 38/E-L 35 “Noble” genes	Proteasome	7

In the berries of “Noble,” we found genes for fatty acid metabolism and degradation. Fatty acid and lipid signaling play roles in resistance to fungal and oomycete diseases in grapes (Fung et al., 2008). Molecular investigation of these fatty acid pathways would assist in determining the mechanisms of tolerance to biotic and abiotic stresses in muscadine grape. Interestingly, we found that circadian rhythm pathways were enriched in “Noble” at the onset of ripening, which suggests that berry maturation in certain cultivars could respond to specific circadian oscillations, such as temperature, day length, and light conditions. In the mature berry, we found that pathways involved in the biosynthesis of amino acids, biosynthesis of secondary metabolites, and ribosome proteins were enriched, which suggests that nutrients, flavor, and aroma compounds were produced during the late stage of ripening (Table 2).

### Validation of expression of key gene regulatory pathways

Quantitative PCR was carried out to validate the differential expression of genes observed in the transcriptomic data. Based on the observed changes in expression levels and patterns at the different developmental stages (E-L 32, E-L 35, E-L 38), 15 genes were selected for confirmation. We included a number of cell wall-associated genes, namely a cellulose synthase (E-L 32C1, *VIT_10s0003g01560*), a COBRA-like protein (E-L 32C2, *VIT_14s0083g01150*), a cellulose synthase-like protein (E-L 32C3, *VIT_11s0037g00530*), xyloglucan (E-L 35 N3), endoglucanase 15 (E-L 32 N1, *VIT_13s0067g01200*), and pectin esterase (E-L 35C5, *VIT_11s0016g00330*); a fruit development related gene, namely an ovate-domain containing protein (E-L 32C4, *VIT_06s0004g07900*); some pigment- and flavor-related genes, namely a cinnamoyl-alcohol dehydrogenase (E-L 32 N2, *VIT_04s0044g00190*), a chalcone synthase (E-L 35 N4, *VIT_16s0022g01190*), a mannan endo-1,4 beta mannosidase (E-L 38 N5, *VIT_18s0089g00170*), and a flavonoid-3 monooxygenase (E-L 38 N6, *VIT_06s0009g02920*), as well as several uncharacterized genes (E-L 35C6, *VIT_02s0025g00320;* E-L 35C7, *VIT_01s0010g03060;* E-L 38C8, *VIT_06s0004g05380;* E-L 38C9, *VIT_06s0004g01300*). The gene names and primer sequences are listed in Supplementary Table 6. The qPCR expression patterns of selected genes displayed similar trends to the transcriptome data (Supplementary Figure 4). Transcript analysis revealed two patterns. One set of transcripts showed a steep increase during the ripening phase, while another set of transcripts showed an increase from the bunch-closure phase to veraison followed by a gradual decrease during the mature phase (Supplementary Figure 4). The transcript levels of cellulose synthase significantly increased at maturity in “Carlos,” but in “Noble” showed enhanced expression from bunch closure to veraison followed by the gradual arrest of transcriptional activity during ripening. Pectinesterase functions in cell wall metabolism to help fruits to ripen by changing the texture of fruits and vegetables. Major changes in coloration and in the cell wall structure occur during the transition from veraison to maturity. Primary cell wall biosynthetic enzymes (list which of those above represent this) and phenolic metabolism (list which of the 15 genes represent this) were active during muscadine ripening. Hence, Pectinesterase is less involved during bunch-closure and more active during the maturation phase in both cultivars (Supplementary Figures 4B,D,F,I).

## Conclusion

**Figure 6:**
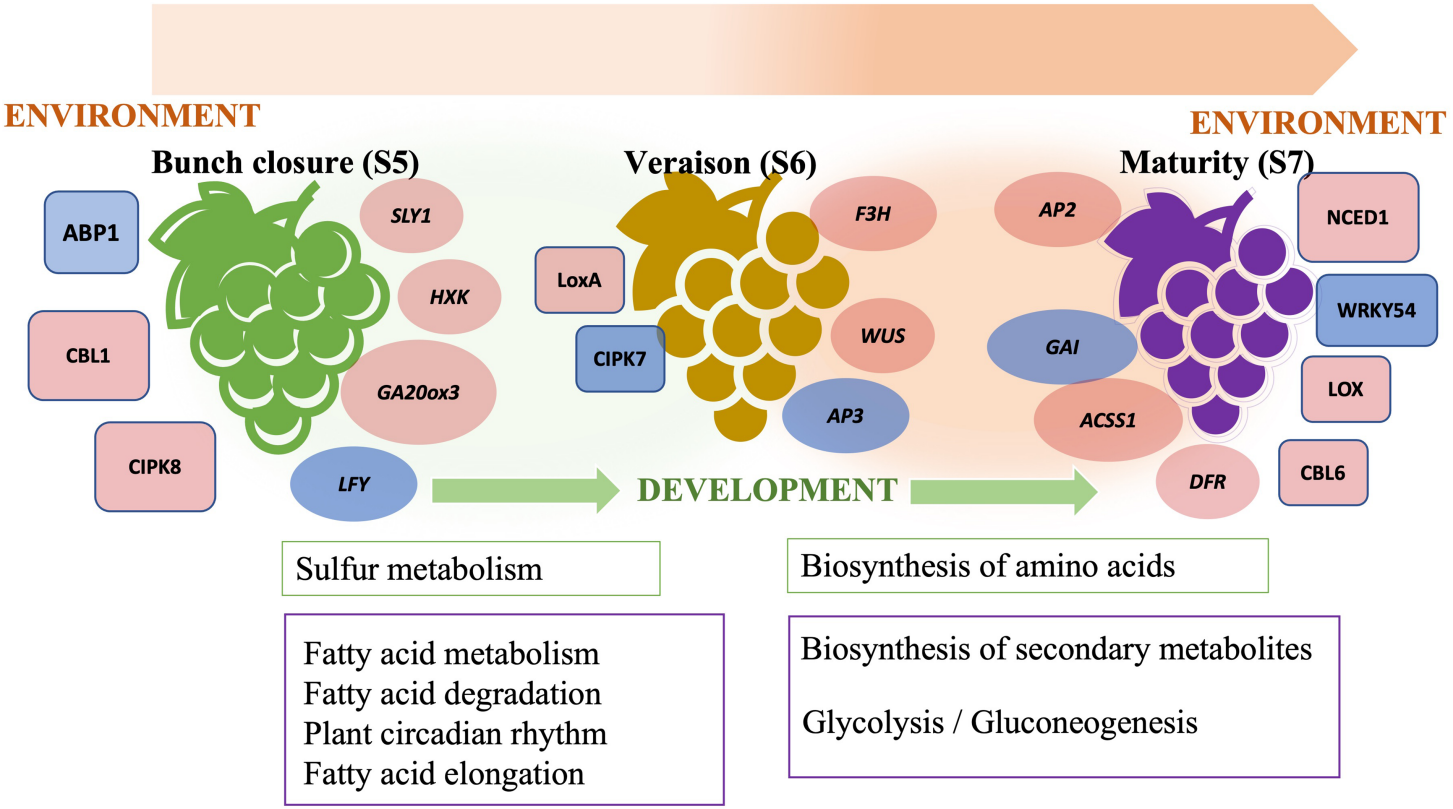
The schematic graph of berry ripening in muscadine grape.

We have described a transcriptome analysis of muscadine grape berries at the final three stages of ripening, namely bunch closure, veraison, and maturity, in two cultivars, “Carlos” and “Noble.” Bunch closure is an early stage of berry growth, which involves fruit expansion, seed growth, and chemical transitions. Veraison is characterized by fruit enlargement and pigment development. Maturity is the final stage of berry ripening that is characterized by changes in nutrients, flavor, and aroma. Those changes heavily influence the postharvest quality and composition of the berries. The transition from veraison to ripeness is the final stage of grape development. Bunch closure stage was identified as the beginning of berries touching. At this stage, the berries were still green and hard, but they started to soften and accumulate sugar. At veraison stage, the berries continued softening and accumulating sugar, and they began to color and enlarge. At maturity stage, they are soft and sweet enough for consuming and fully colored (Coombe, 1995; Carbonell-Bejerano et al., 2016; Ghaffari et al., 2020). This analysis demonstrated genetic differences between the two cultivars and identified ripening-stage specific changes in gene expression. Differentially expressed genes included regulatory network components and metabolic pathways. These findings corroborate the previously described “switch genes” active in vegetative-to-mature transition (Palumbo et al., 2014). We also identified additional transcriptional regulators, developmental and environmental responsive gene networks, and metabolic pathways during fruit ripening (Figure 6). These results increase our knowledge of the molecular mechanisms underlying grape berry ripening, including changes in the expression of cell wall modifying enzymes throughout the three ripening phases. This strengthens the idea that grape berry softening during ripening is associated with changes in cell wall polysaccharides in pericarp and mesocarp tissues.

The onset and progression of ripening in grape berry is regulated by both developmental and environmental factors that are signaled through plant hormones and stress-responsive pathways. Plant hormone cross-talk is critical for ripening (Figure 6). GA-responsive genes are crucial during seed and fruit development in muscadine grape. In grapevine, it has been well-studied that GA application leads to berry enlargement and seed development in seeded cultivars (Acheampong et al., 2017). This suggested that further exploration of GA signaling in muscadine grape is needed for the induction of seedless berries and in breeding new varieties. Additionally, cross-talk between hormone response pathways permits fine-tuning of plant growth and development in response to various environments. We further demonstrated the importance of JA and ABA biosynthesis and signaling genes during ripening. Characteristic features of muscadine grapes are that they are a sustainable fruit crop suitable for growth in the southeastern United States and that they are tolerant of various abiotic and biotic stresses (Andersen et al., 2010). This study illustrated the importance of JA- and ABA-regulated stress signaling pathways. Interestingly, we found that karrikin signaling genes were activated during early ripening. Although karrikins are mainly involved in the release of seed dormancy, the regulation of seed germination, and the establishment of seedlings through mimicking the function of strigolactone (Waters et al., 2014), it is worth noting that karrikins may also play a role in fruit ripening.

This study also highlighted the genetic variability and diversity between the “Carlos” and “Noble” cultivars. Genes encoding wax and cuticle biosynthesis and metabolism were differentially expressed at the early stage of ripening in “Carlos” (Figure 5). Cuticular wax is known to accumulate during berry development in response to stresses (Dimopoulos et al., 2020). It is known that the wax content of the grape berry surface is closely related to fruit glossiness, skin thickness, and postharvest shelf-life (Zhang et al., 2021). One major undesirable characteristic of muscadine grape is the thick, leathery skin compared to table grape (Andersen et al., 2010). These results provide a good foundation for exploring the genes related to cuticle wax biosynthesis and how they can be manipulated to reduce the leathery skin in muscadine grape. Our transcriptional analysis during berry ripening determined that transcription factors play important roles in determining the phenotypic differences between these two cultivars.

Muscadine grapes are commonly grown in southeastern of the United States. A few ongoing breeding programs actively breed muscadines for both fresh and value-added markets in the region. Berry traits such as composition, texture, flavor, and aroma are important attributes for consumer acceptance of muscadine grapes. Our quantitative transcriptome analysis (RNA-seq) of “Carlos” (bronze) and “Noble” (black) muscadine cultivars was able to identify genes that may control berry phenotypes, such as the accumulation of high levels of antioxidants. The reference transcriptome developed in this study can be used by muscadine breeders to identify candidate genes for antioxidant biosynthesis, berry firmness, texture, skin thickness, seedlessness, and flavor/aroma, as well as improved vines for tolerance to both biotic and abiotic stresses (e.g., cold hardiness, diseases, and insects resistance).

## Data availability statement

The original contributions presented in the study are publicly available. This data can be found at: NCBI, GES203347.

## Author contributions

AS and TL conceived the experiment(s). YC and YA conducted the experiment(s). TG and TL analyzed the results. TL, AS, and YC wrote and reviewed the manuscript. All authors contributed to the article and approved the submitted version.

## Funding

This work was supported in part by funds from the National Institute of Food and Agriculture, USDA-NIFA (P0204625 for TL).

## Conflict of interest

The authors declare that the research was conducted in the absence of any commercial or financial relationships that could be construed as a potential conflict of interest.

## Publisher’s note

All claims expressed in this article are solely those of the authors and do not necessarily represent those of their affiliated organizations, or those of the publisher, the editors and the reviewers. Any product that may be evaluated in this article, or claim that may be made by its manufacturer, is not guaranteed or endorsed by the publisher.

## Footnotes

^1^ http://www.bioinformatics.babraham.ac.uk/projects/fastqc/

^2^ http://hgdownload.soe.ucsc.edu/downloads.html#human

^3^ https://string-db.org/

^4^ http://plantregmap.gao-lab.org/go.php

^5^ http://plantregmap.gao-lab.org/tf_enrichment.php

^6^ http://plantregmap.gao-lab.org/tf_enrichment.php

^7^ http://stitch.embl.de

## Supplementary material

SUPPLEMENTARY FIGURE 1 GO-term enrichment analysis of the E-L 32-, E-L 35-, and E-L 38-specific genes in ‘Carlos’ in the category of (a) biological process, (b) cellular component, and (c) molecular function.

SUPPLEMENTARY FIGURE 2 GO-term enrichment analysis of the E-L 32-, E-L 35-, and E-L 38-specific genes in ‘Noble’.

SUPPLEMENTARY FIGURE 3 GO-term enrichment analysis of the switch genes in early stage of ripening in ‘Carlos’.

SUPPLEMENTARY FIGURE 4 Relative expression levels of transcripts changes in ‘Carlos’ and ‘Noble’ muscadine grapes.

SUPPLEMENTARY FIGURE 5 Genes expression atlas in grape developmental data source Grape eFP Brower at bar.utoronto.ca.

SUPPLEMENTARY TABLE 1 E-L 32-specific, E-L 35-specific, and E-L 38-specific genes list and GO-term enrichment analysis in ‘Carlos’.

SUPPLEMENTARY TABLE 2 E-L 32-specific, E-L 35-specific, and E-L 38-specific genes list and GO-term enrichment analysis in ‘Noble’.

SUPPLEMENTARY TABLE 3 E-L 32-specific, E-L 35-specific, and E-L 38-specific list of genes in both cultivars.

SUPPLEMENTARY TABLE 4 Switch genes analysis in *V. vinifera* and two *M. rotundifolia* cultivars.

SUPPLEMENTARY TABLE 5 RNA-seq data were grouped into nine different clusters using *k*-mean analysis.

SUPPLEMENTARY TABLE 6 Gene names and primer sequences for qPCR.

SUPPLEMENTARY TABLE 7 Read mapping summary.
