# Supplementary figures and images for: Transcriptional profiling of two muscadine grape cultivars “Carlos” and “Noble” to reveal new genes, gene regulatory networks, and pathways that involved in grape berry ripening
Source: Front Plant Sci. 2022 Aug 11;13:949383. doi: 10.3389/fpls.2022.949383 (PMC9435441; doi:10.3389/fpls.2022.949383)

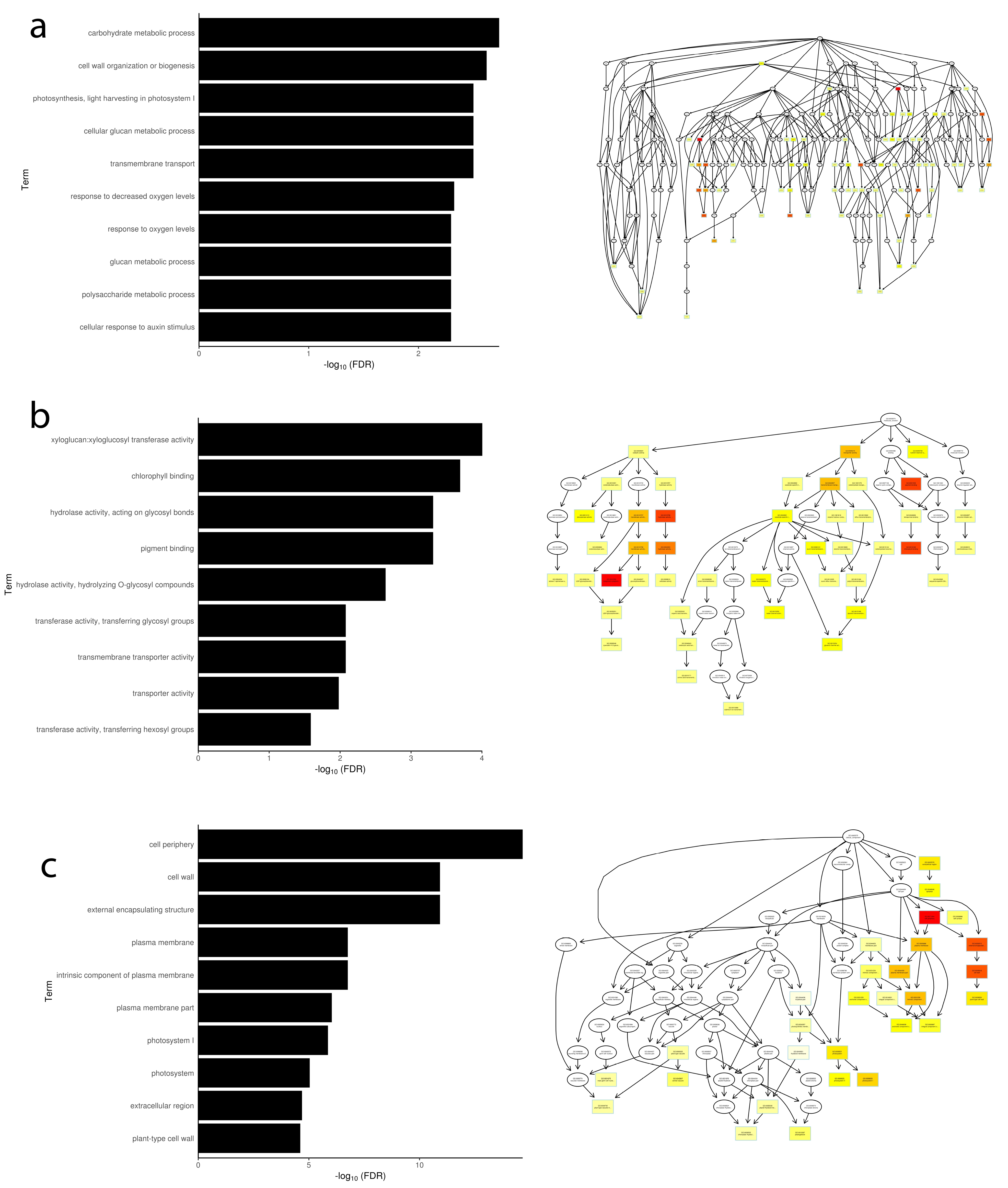

Supplement: SUPPLEMENTARY FIGURE 1 — GO-term enrichment analysis of the E-L 32-, E-L 35-, and E-L 38-specific genes in ‘Carlos’ in the category of (a) biological process, (b) cellular component, and (c) molecular function. [file Image_1.JPEG]

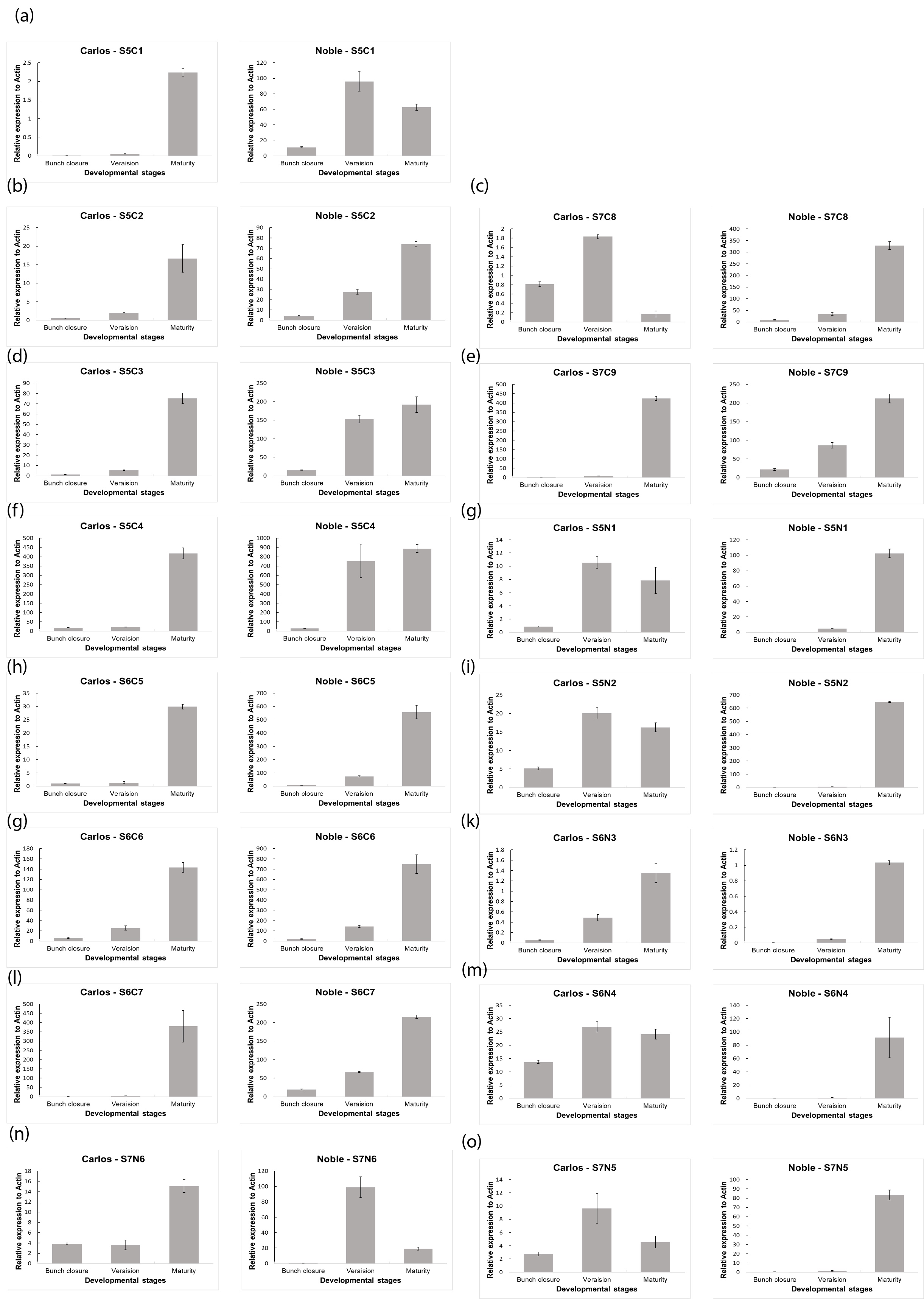

Supplement: SUPPLEMENTARY FIGURE 4 — Relative expression levels of transcripts changes in ‘Carlos’ and ‘Noble’ muscadine grapes. [file Image_4.JPEG]

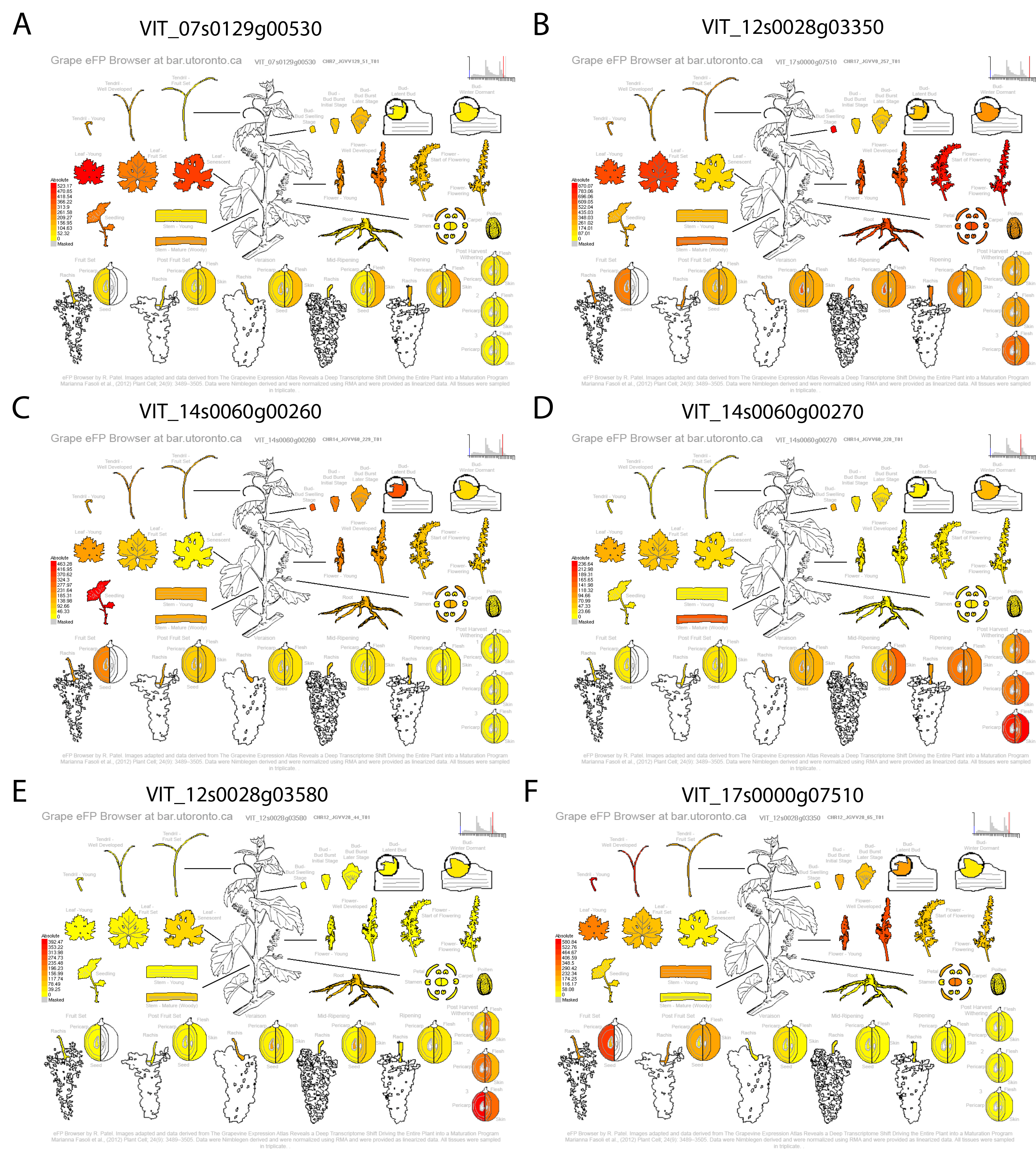

Supplement: SUPPLEMENTARY FIGURE 5 — Genes expression atlas in grape developmental data source Grape eFP Brower at bar.utoronto.ca. [file Image_5.JPEG]
