# Supplementary material for: Transcriptional profiling of two muscadine grape cultivars “Carlos” and “Noble” to reveal new genes, gene regulatory networks, and pathways that involved in grape berry ripening
Source: Front Plant Sci. 2022 Aug 11;13:949383. doi: 10.3389/fpls.2022.949383 (PMC9435441; doi:10.3389/fpls.2022.949383)
Supplement: SUPPLEMENTARY TABLE 6 — Gene names and primer sequences for qPCR. [file Table_6.DOCX]

**Primer used in RT-PCR for the RNA seq validation**

| **NAME** | **SEQUENCE** |
| --- | --- |
| VvS5C1_FP | GAGTTCAAAGTGCGGGTAAATG |
| VvS5C1_RP | GGGTGGTCACGTGGATTATT |
| VvS5C2_FP | CAGTCCTTTCCCGCTATTCTT |
| VvS5C2_RP | GTGTAGAGTTAGGAGATGGCTTAC |
| VvS5C3_FP | GGGCTGTCATCTTCACTTCATA |
| VvS5C3_RP | CCAGTCATCCATTCGCTCTT |
| VvS5C4_FP | TCCGTTTCCAAAGGCAAGA |
| VvS5C4_RP | GGTGTAAGAGGTGATGGGAATAG |
| VvS6C5_FP | GGGTCACTCCAAGGTTGATATT |
| VvS6C5_RP | GCCAACTGGTAGCTGAATTTG |
| VvS6C6_FP | GGCACCTCTAGCATCATCTATAAC |
| VvS6C6_RP | CAGGCTGCTTCATCATCAAATC |
| VvS6C7_FP | CCCAAATGTCTCTGATGCCTAT |
| VvS6C7_RP | CTTTCCGGGCTTCACCTTTA |
| VvS7C8_FP | CTGGATTAGGAGCAACCATACA |
| VvS7C8_RP | TCACACACTGAACCACTCAC |
| VvS7C9_FP | GATGGTCTCTCAAGGGAATGAC |
| VvS7C9_RP | CGTGTGTATGGTTGCTCCTAA |
| VvS5N1_FP | CGGTCCAGAAGGGTTACAATAA |
| VvS5N1_RP | CGTAGGAGATTGTGAGGAGTAAAG |
| VvS5N2_FP | CAAGATGGGACGAGGACTTATG |
| VvS5N2_RP | CCTGCATCTAAGGGCATGTTA |
| VvS6N3_FP | CTCTTCACTTCCACGCTCAA |
| VvS6N3_RP | GAGCAGAAGAGAGACCAAGAAC |
| VvS6N4_FP | CTGCCCTCAGATTGGCTAAA |
| VvS6N4_RP | GGCTTACGGAAAGTCGATGT |
| VvS7N5_FP | CGACCCAAGCCAAAGGAATA |
| VvS7N5_RP | GAAGAGCTTGATTGCCTCCA |
| VvS7N6_FP | AAAGGGTCGGAGTCAAATGAG |
| VvS7N6_RP | CCATGCGATGGACGGAATAA |
